# Spinal tuberculosis: diagnosis and management

**Published:** 2012-11

**Authors:** Mohammad Rasouli, Maryam Mirkoohi, Alexander R. Vaccaro, Kourosh Karimi Yarandi, Abtin Shahlaee, Vafa Rahimi-Movaghar

**Affiliations:** ^a^Sina Trauma and Surgery Research Center, Tehran University of Medical Sciences, Tehran, Iran.; ^*b*^The Rothman Institute of Orthopaedics, Thomas Jefferson University, Philadelphia, PA, USA.; ^*c*^Research Centre for Neural Repair, Tehran University, Tehran, Iran.

## Abstract

The involvement of spinal column reportedly occurs in less than 1% of all tuberculosis (TB) patients. Spinal TB is a very dangerous type of skeletal TB as it can be associated with neurologic deficit because of compression of adjacent neural structures and significant spinal deformity. Therefore, the early diagnosis and management of spinal TB has special importance to prevent these serious complications. Therefore, the present study was aimed at conducting a comprehensive narrative review and analysis of all the papers available for us published during 1990 to 2011 to extract current trends in diagnosis and medical or surgical treatment of spinal TB. Although the development of more accurate imaging modalities such as magnetic resonance imaging (MRI) and advanced surgical techniques have made the early diagnosis and management of spinal TB much easier, these are still very challenging topics. In this review we aimed to discuss diagnosis and management of spinal TB based on the studies with acceptable design, clearly explained results and justifiable conclusions.

**Keywords:**

Spinal tuberculosis, Diagnosis, Treatment


					Volume 4, Suppl. 1 Nov 2012
Publisher: Kermanshah University of Medical Sciences
URL: http://www.jivresearch.org
Copyright:
Congress of Iranian Neurosurgeons, 14-16 November 2012, Kermanshah, Iran
Paper No. 43
Spinal tuberculosis: diagnosis and management
Mohammad Rasouli a,b
, Maryam Mirkoohi a
, Alexander R. Vaccaro b
, Kourosh Karimi Yarandi a
,
Abtin Shahlaee a
, Vafa Rahimi-Movaghar a,c,*
a
Sina Trauma and Surgery Research Center, Tehran University of Medical Sciences, Tehran, Iran.
b
The Rothman Institute of Orthopaedics, Thomas Jefferson University, Philadelphia, PA, USA.
c
Research Centre for Neural Repair, Tehran University, Tehran, Iran.
Abstract:
The involvement of spinal column reportedly occurs in less than 1% of all tuberculosis (TB) patients. Spinal TB is a very
dangerous type of skeletal TB as it can be associated with neurologic deficit because of compression of adjacent
neural structures and significant spinal deformity. Therefore, the early diagnosis and management of spinal TB has
special importance to prevent these serious complications. Therefore, the present study was aimed at conducting a
comprehensive narrative review and analysis of all the papers available for us published during 1990 to 2011 to
extract current trends in diagnosis and medical or surgical treatment of spinal TB. Although the development of more
accurate imaging modalities such as magnetic resonance imaging (MRI) and advanced surgical techniques have made
the early diagnosis and management of spinal TB much easier, these are still very challenging topics. In this review
we aimed to discuss diagnosis and management of spinal TB based on the studies with acceptable design, clearly
explained results and justifiable conclusions.
Key words:
Spinal tuberculosis, Diagnosis, Treatment
* Corresponding Author at:
Vafa Rahimi-Movaghar: Associate professor of Neurosurgery, Research Deputy, Sina Trauma and Surgery Research Center, Sina Hospital,
Hassan-Abad Square, Imam Khomeini Ave, Tehran University of Medical Sciences, Tehran, Iran. Phone: (+98) 915 342 2682, (+98) 216 6757010,
Fax: (+98) 216 675 7009, Email: v_rahimi@sina.tums.ac.ir , v_rahimi@yahoo.com, (Rahimi-Movaghar V.).

				

